# Current Challenges in Environmental Decontamination and Instrument Reprocessing

**DOI:** 10.1016/j.identj.2024.08.016

**Published:** 2024-11-06

**Authors:** Laurence J. Walsh

**Affiliations:** The University of Queensland School of Dentistry, UQ Oral Health Centre, Herston, Queensland, Australia

**Keywords:** Decontamination, Cleaning, Disinfection, Sterilisation, Ultraviolet C, Hydrogen peroxide

## Abstract

Decontamination of the clinical working environment and reprocessing of reusable dental instruments and devices are key components of modern infection control. This narrative review, which is part of a special issue devoted to contemporary infection control practices, highlights the latest evidence and the potential role of emerging technologies. The underpinning concepts of environmental decontamination and reprocessing have remained unchanged for many years, and key principles such as cleaning before disinfection or sterilisation remain true to the present day. What has changed in recent years are the range of options available, most notably for no-touch decontamination as an adjunct to regular environmental cleaning methods, including vapourised hydrogen peroxide, hydroxyl radicals, and ultraviolet C irradiation. In the realm of sterilisation, newer approaches include solar autoclaves and hydrogen peroxide gas plasma sterilisation. With all new and emerging technologies, greater attention is now being paid to safety as well as effectiveness, with stronger consideration of environmental impacts. This is especially relevant to the use of fully disposable surgical instruments vs reusable instruments. Because dental clinics have many configurations and sizes, each clinic needs to undertake local risk assessments to inform their decisions regarding suitable decontamination and reprocessing methods.

## Introduction

Decontamination activities form a central part of modern infection prevention and control. This paper focusses on recent developments in the decontamination of environmental surfaces and dental instruments and highlights the latest evidence and the place of emerging technologies.

One must also be aware that new and emerging infectious diseases could prompt reassessments of methods, materials, and devices used for decontamination of the clinical working environment and for instrument reprocessing, based on the challenges posed by new pathogens. As an example, once the nature of the pathogen responsible for COVID-19 was known (ie, an enveloped virus), it was possible to determine suitable agents for surface management by using surrogate viruses, even when the pathogen itself was not readily available to be used in such experiments, as the inactivation characteristics of enveloped viruses are well known.^1^

## Environmental surface management

### Cleaning

Physical cleaning is an essential requirement for environmental surfaces and a prerequisite for effective disinfection and sterilisation. For the surfaces of dental equipment and benchtops within dental clinics, a common approach is to use wipes where the matrix is impregnated with a neutral or slightly alkaline detergent. It is important that the wipe ingredients do not cause the surface to degrade, as this would make that surface harder to clean and disinfect over time and ultimately worsen its appearance. In decreasing order of difficulty of cleaning, dental chair surfaces can be ranked from textured vinyl (the most difficult), to smooth vinyl, enamelled metal, tubing, and brushed aluminium.^2^

A major challenge is determining whether physical cleaning has been thorough and effective. Direct visual examination lacks sensitivity but is simple to undertake and gives some level of immediate feedback. Visual inspection is a poor indicator of cleaning performance when compared with microbial culture methods, fluorescent dye indicators, or ATP bioluminescence.^3^, ^4^, ^5^ Whilst ATP assessment requires a special portable meter, fluorescent “soiling spots” can be detected using low-cost light-emitting diode (LED) torches that emit light in the ultraviolet-A to visible violet range (350–430 nm). Fluorescent powders and spots can be placed on benchtops and on high-touch surfaces or objects before cleaning, and their presence assessed after cleaning, to rate the overall effectiveness of the cleaning process in a qualitative manner.

Traditional wipes are made from polypropylene, and their manufacture and disposal generate microplastics, which are problematic. A range of alternative materials have been developed that are not affected by this issue, including reusable wipes made from biodegradable bamboo fibres that can be laundered and then used again and wipes made from bagasse and other plant-based materials that undergo biodegradation when in a landfill.

### Disinfection after cleaning

Disinfectants are used either following cleaning or simultaneously in combination with detergents to manage a range of surfaces and shared devices, according to level of risk. The most widely used disinfectant products are classified as low-level and contain quaternary ammonium compounds and phenolics as active ingredients. Such products are designed not only to be effective for decontamination but also to have an acceptable safety profile. Issues in staff members such as asthma and contact allergies from occupational exposure are exceedingly rare when proper personal protective equipment is worn when these products are being used to wipe down surfaces.^6^

A key issue with all disinfectants is their use profile in terms of interactions with surfaces being cleaned, spectrum of activity, end user health effects, and environmental impacts. These various aspects need to be optimised whilst trading off undesirable characteristics with effectiveness, cost, and shelf life. Examples of compatibility issues include iodophors causing staining of some plastic polymer materials, certain phenolics causing degradation of plastic polymers, primary alcohols causing upholstery to degrade, and some quaternary ammonium compounds leaving a surface residue. Agents such as chlorhexidine that have limited effectiveness against many Gram-negative bacteria need to be combined with other biocides that are broad-spectrum; however, it is not possible to combine chlorhexidine with oxidants because of degradation of the chlorhexidine into para-chloroaniline. The shelf life of some biocides based on NaOCl or HOCl is strongly influenced by temperature and pH, which creates issues for their use in warm climates. Finally, biocides entering the waste stream vary in their environmental impacts. Hydrogen peroxide degrades to water and oxygen, creating no adverse impacts, whilst other biocides may persist in the environment and affect plant, aquatic, and animal life.

### Environmental impacts of barriers

The use of barriers on items of equipment must be dictated by the manufacturer's instructions for use (IFU) for those items that cannot tolerate routine cleaning, disinfection, and sterilisation. Many dental chairs are made with surfaces designed for regular wiping with common types of detergent-based wipes, thus removing the need for barriers, regardless of whether the patient is healthy, immunocompromised, or known to carry an infectious agent. Barriers should not be placed onto dental equipment “by habit,” but when there is a clear rationale and a directive from the manufacturer as to what barrier materials are suitable. This prevents excessive use of barriers in the dental clinic setting.

Plastic barriers can represent a significant input into the waste stream of a dental practice. A range of biodegradable plastic barriers has become available in recent years. Increasing the complexity around this topic is inconsistent labelling regarding the features of these barriers, including “greenwashing,” wherein product labelling is misleading. A further concern is that some barrier materials may generate microplastic particles as they undergo degradation. Label claims must refer to the relevant ISO standards for biodegradation of polymers, including aerobic biodegradation in water (ISO 14851, 14852), anaerobic biodegradation in water (ISO 14853), aerobic composting (ISO 14855), anaerobic high solid decomposition (ISO 15985), aerobic soil burial (ISO 17556), and disintegration at a pilot scale (ISO 16929).

### New developments in the management of environmental surfaces

Over recent years, interest in no-touch technologies to enhance decontamination of surfaces in clinical working areas has grown. Evidence has shown that adjunctive use of these technologies can reduce contamination remaining on surfaces after standard cleaning methods. Each no-touch approach has practical issues including cost, simplicity of operation, ease of use, time required to complete decontamination cycles, and safety for staff in the area being treated.^7^

### Vapourised hydrogen peroxide

Since the COVID-19 pandemic, the use of vapourised hydrogen peroxide (VHP) has become more common. Once dental operatories have been physically cleaned by wiping using a suitable detergent-based product, VHP can be used to disinfect surfaces. Staff need to leave the room whilst the VHP unit is generating the vapour fog. The vapour from 35% hydrogen peroxide will spread throughout the operatory by normal air movements in the room and will reach areas that are difficult to access. VHP is effective against all pathogens encountered in the dental clinical setting^8^ and has been shown to be highly effective for eradicating persistent environmental contamination by resistant pathogens.^9^

Further research is needed into how the performance of VHP in a dental clinic setting varies according to the room air-handling arrangements (eg, air changes per hour and type of air conditioning system). VHP systems typically can monitor the hydrogen peroxide concentration that is being achieved (eg, in parts per million) to track levels over the vapour treatment period, enabling staff to check these readings.

The vapour leaves no harmful residues, as the breakdown products are water and oxygen. However, if staff enter the room whilst the VHP fogger is still in operation, they may experience irritation in their eyes as well as in their respiratory tract from inhaling the vapour. Hence, it is important to follow the manufacturer's advice regarding how long is needed after operating the VHP cycle to ensure that proper venting has occurred before staff recommence work and set up for a new patient. This period of work interruption from a single VHP cycle can be several hours.

### Hydroxyl radicals

Unlike ozone generators or VHP foggers, hydroxyl radicals (OH˙) are less likely to cause respiratory irritation, which increases the possibility that a system that generates such radicals could operate whilst a dental clinic was running normally. Preliminary positive results have been reported from using hydroxyl radicals to reduce the levels of total aerobic bacteria on the surfaces of a dental chair.^10^

A limitation of this technology is that Gram-positive bacteria, because of their thicker cell wall, will be less vulnerable to attack than Gram-negative bacteria. A 2023 systematic review identified 8 laboratory studies that supported the efficacy of hydroxyl radical disinfection and highlighted the speed of action and low toxicity as advantages over VHP and ozone for wide-area surface disinfection.^11^ This method may have value when used as a supplement to conventional surface cleaning and disinfection methods.

### Ultraviolet C irradiation

The highly antimicrobial actions of ultraviolet-C (UVC) radiation from 200 to 280 nm have been known for more than 100 years, and various UVC systems have been deployed in hospital settings (including in operating theatres).^12^, ^13^, ^14^ The COVID-19 pandemic sparked interest in deploying this technology in dental clinics and in other health care settings.^15^^,^^16^ UVC light can be generated from lamps (such as mercury low-pressure vapour lamps emitting at 254 nm) and from LEDs (emitting at 222 nm). Importantly, UVC radiation causes only “line-of-sight” inactivation of microorganisms on surfaces by changes in DNA that interfere with replication. UVC irradiation creates pyrimidine dimers, causing adjacent bases to bond to one another, instead of bonding across the DNA double helix.^17^^,^^18^

In a 2022 systematic review, most of the 12 included studies reported a 1-log to 2-log reduction in bacterial levels on surfaces in 10 to 25 minutes when UVC was used, and 3 studies reached a 5-log or 6-log reduction.^19^ Correct positioning of UVC lamps is essential because any surfaces that are not in the direct line of sight but are in a shadow zone will not be effectively treated. The time needed for disinfection will vary according to the power of the UVC light source and the distance from the light source to the target surface.

UVC radiation at a wavelength of 254 nm is generated by low-pressure mercury vapour lamps. Emissions from such lamps with high levels of exposure can cause acute photodermatitis as well as photokeratoconjunctivitis.^20^^,^^21^ For this reason, 254-nm mercury lamps are not suitable for continuous use in occupied working areas; however, they can be used after working hours when no staff are present. Accidental exposure to 254-nm UVC radiation can be prevented by using passive infrared proximity sensors to shut down lamps when people enter the area.

Mercury vapour lamps have a shorter life-span than LEDs, with annual replacement the norm, and used lamps need special attention at the end of their working life, as they contain mercury. Local regulations for disposal must be followed. On the other hand, 222-nm light can selectively inactivate microorganisms, including human influenza viruses, and coronaviruses, as well as bacteria,^22^ but does not damage human cells, since 222-nm light is unable to penetrate the stratum corneum of the skin.^23^

Long-term data on safety with 222 nm are incomplete, and so it remains unclear how long staff can be working in a clinic whilst 222-nm LED lights are operating. Hence, this aspect needs further evaluation.^24^ Another issue with continuously operating UVC light sources is the extent of photodegradation that occurs to nonmetallic surfaces in the working environment, especially those made from plastic polymers.^25^

## Instrument cleaning

### The Spaulding classification

As mentioned earlier, both disinfection and sterilisation methods will fail if instruments are not cleaned effectively and viable microorganisms or clinical soil remains. The modern approach to reprocessing of instruments follows the classification developed by Dr E.H. Spaulding more than 50 years ago,^26^ with items being classified as critical (where a device or item enters sterile tissue and hence must be sterilised and then maintained in a sterile state until used), semicritical (where a device or item contacts mucous membranes or non-intact skin and requires high-level disinfection where sterilisation is not possible), or noncritical (where a device or item comes into contact with intact skin and requires cleaning and, in some cases, also low-level disinfection).

This approach remains valid in 2024^27^; however, some refinements can be made. For example, the noncritical category can be divided further to differentiate between surfaces that come into direct contact with the patient's skin (eg, blood pressure cuffs); high-touch surfaces that could serve as fomites for the transfer of micro-organisms (eg, keyboards, door handles); and minimal-risk surfaces (eg, floors).^28^^,^^29^ Another suggested refinement is to include within the critical item definition “objects which directly or indirectly (ie, via a mucous membrane) enter normally sterile tissue of the vascular system or through which blood flows should be sterile.”^30^

### Precleaning and holding solutions

Correct precleaning of instruments involves removing gross soil. This should be done at the point of use to prevent materials drying onto the surface. In addition, poor instrument management prior to cleaning can increase the complexity of the cleaning process. Examples of poor practices include soaking in water for prolonged periods, which provides sufficient time allows biofilms to form, and wiping or spraying instruments with ethanol or isopropanol, which will fix proteins onto stainless steel surfaces.^31^ On the other hand, placing instruments in presoaking solutions designed for this purpose overcomes concerns when there is a delay between the end of a procedure and the start of reprocessing. Under such conditions, long holding times do not lead to greater protein residues on instruments or cause greater corrosion.^32^

### The importance of the IFU

Instrument cleaning must follow the advice provided by the manufacturer in their IFU. These reprocessing instructions should describe, in detail, the individual steps, including disassembly and lubrication where these are needed. Clinical staff should consider the reprocessing requirements when purchasing new items, as this will determine whether the clinic has the necessary equipment (eg, if a washer disinfector is specified for cleaning).

Staff need to carefully read the IFU for cleaning products and pay attention to advice regarding appropriate use (including dilution ratios, and suitability for use in ultrasonic cleaners) and safe handling precautions. Occupational issues can arise if staff are not aware of risks caused by products with highly alkaline pH (eg, pH >10.5) or those containing proteolytic enzymes, which can cause skin irritation. Compatibility issues are also important to identify, including alkaline products that will cause corrosion of aluminium instruments.^33^

### Workflow

A key concept in instrument reprocessing is having a unidirectional flow of items from dirty to clean. Ideally, there should be airflow in the opposite direction, from clean to dirty, to limit the possibility of contamination occurring between different zones within the reprocessing area. For the same reasons, instrument reprocessing should not be performed in the dental operatory itself, but in a physically segregated area, as instrument reprocessing activities contaminate the area where they are performed.^34^ Physical separation also ensures that sterilised items are not contaminated by splashes of fluid from patient procedures.

## Instrument sterilisation

Local regulations dictate the level of information that is recorded about each steriliser cycle. Checking the steriliser printout is just one step in the process of checking that a load of instruments has been effectively sterilised. The steriliser printout (or digital record) is a physical indicator that indicates whether the stated cycle parameters have been reached. This record is used in combination with chemical indicators to show that certain temperatures, times, and steam exposure conditions have been reached during the sterilising process. Provided that cycle parameters have been achieved and the load items have been exposed to the correct parameters (as shown by the results of the physical and chemical indicators), once items have been inspected to verify the integrity of the packaging, wrapped sterilised instruments can be released for use in the clinic. This is known as parametric release and must be recorded and signed off by the person who is performing the task.

Key items to check for include that all packages are dry and are free of damage (ie, intact seals and no items penetrating through the packaging), that seals are intact along their length with no interruptions, and that any external and internal chemical indicators have undergone the required colour change. An example of a comprehensive cycle record for an individual steam steriliser cycle is shown in the Table,^35^ which shows the steps involved in checking a sterilised load. Each separate steam steriliser used in a clinic needs to have cycle records.

**Table:** An example of data fields collected for a steam steriliser cycle record.

Entered before starting the cycle	Entered at the end of the cycle prior to load release
Date and time	Check of data/printout of cycle parameters
Cycle number and batch code	Check of chemical indicators
Cycle type selected	Check for package integrity including seals
Summary of the load contents	Check for package dampness
Loading operator identification	Unloading operator identification

It is essential that staff remove from circulation and quarantine any packages with defects or for which the chemical indicators reflect insufficient exposure to steam, as these represent the results of flaws in the steam sterilising process, such as operator error, a malfunctioning steriliser (eg, a leaky door seal), and poor steam quality due to poor-quality deionised water. Common operator errors include incorrect cycle selection, inappropriate packaging materials or packaging techniques, incorrect loading of the chamber, and incorrect use and interpretation of chemical indicators.^36^ All sterilisation process failures must be investigated so that the root causes of the failure can be identified. Any nonconforming defective packages that are found must not be used in the clinic for patient care.

Whilst there are international (ISO) standards for the use of chemical indicators and biological indicators during sterilisation, the application of these varies around the world. In some jurisdictions, an annual validation is undertaken using thermometric tests and biological indicators in 3 consecutive cycles for the most challenging load item. This provides the platform for choosing appropriate sterilisation cycles for the following year. Validation is only repeated when the methods and materials used for packaging change or the complexity of the most difficult load item increases. This approach requires as few as 10 biological indicators per practice per year. At the other extreme are jurisdictions where a biological indicator is used every working day or once every week. This results in a greatly increased usage of such biological indicators, even though there is no evidence that this is superior to the annual validation approach.

### Appropriate storage

Correct packaging of instruments provides a barrier against microorganisms.^37^ High temperature and high humidity cause degradation of the paper component of pouches, whilst direct sunlight can also cause degradation of plastic components of pouches.^38^ This is why pouches containing instruments for use in critical sites (eg, for surgical exodontia) need to be stored away from direct sunlight and not subjected to frequent handling.

### Environmental impact considerations

Instrument cleaning and steam sterilisation use considerable amounts of electrical energy as well as high-quality water for rinsing and deionised water for steam generation. To improve efficiency and conserve valuable resources, clinics should periodically review their instrument handling routines. A particular issue is the unseen costs of reprocessing items within kits of instruments, burs, and the like that are unused. Such costs arise because of the staff time needed for cleaning, inspection, and packaging. In addition, kits or trays with more needed instruments lead to more errors when choosing instruments, as has been shown in operating theatre environments.^39^^,^^40^ Rationalisation of instrument sets should be undertaken, considering the optimal mix for providing safe patient care. Items and instruments that are rarely used should be packaged separately and retrieved when needed, rather than included in every instrument set.

The temptation must be avoided to alter metallic instruments in ways that destroy the passivated surface, as this will make it prone to corrosion. Engraving causes such changes where the surface was broken, and once visible corrosion is present, effective reprocessing can no longer be accomplished and the instrument must be discarded (typically into the sharps waste stream).

### Sterilisation pouches

To date, little work has been done on the concept of recycling of paper-plastic sterilisation pouches. Proper waste stream segregation requires separating the paper component from the plastic component. This needs to be done in such a way that the materials for recycling are kept free from contamination. Ideally, the plastic component could then undergo chemical recycling. This involves breaking the polyethylene (PE) and polypropylene (PP) polymers down by exposing these to high temperatures (200 to 800 °C) in the absence of oxygen to create hydrocarbon oil or gas, which could then be used to rebuild new polymers. At present, such chemical recycling processes are uncommon and account for less than 1% of all plastic recycling.^41^^,^^42^ The environmental impacts of the solvents and chemical processes used in recycling must also be considered.

Reuseable surgical wrapping materials and reuseable paper-plastic pouches are available in some regions, but their use is not commonplace at the global level. Using such materials could considerably reduce the volume of waste generated by dental clinics and lower the environmental impact of a dental clinic.

## New developments in sterilisation

### Solar energy–powered steam sterilisation

Steam sterilisation is used widely for sterilising dental instruments. It has modest costs and high reliability but requires considerable electrical energy to heat deionised water to generate steam. This makes it challenging in remote parts of developing countries, where mains electricity supplies are irregular or nonexistent. This has led to the concept of a solar autoclave that is powered completely by solar radiation, with the radiant energy of sunlight collected and converted to thermal energy for steam generation. For the solar autoclave concept, 5 different types of collectors have been described: parabolic dishes, parabolic trough collectors, Fresnel collectors, evacuated tube collectors, and flat plate collectors. For the first 2 types, some type of tracking system is needed to track the position of the sun in the sky, whilst other designs require no such input.^43^ Some of these designs have no moving parts and can reach sterilising conditions of 121 °C within 40 minutes of operation.^44^

### Hydrogen peroxide gas plasma sterilisation

Hydrogen peroxide gas plasma sterilisation (HPGPS) has been used in large hospitals to sterilise heat-sensitive items since the 1990s,^45^^,^^46^ and in the past 4 years they have entered the dental market in the form of compact benchtop units with integrated or attached vacuum pumps.

HPGPS is suitable for sterilising polymer-based instruments and items that cannot be subjected to steam sterilising because they would melt or degrade. Instruments leave the cycle only slightly above room temperature and can be used immediately. Small-chamber HPGS units have fast cycle times, thus improving turnaround and reducing the need for a large instrument inventory. Their cost is similar to that of steam sterilisers. The hydrogen peroxide is dosed from special cartridges, and there is no requirement for deionised water, as no steam is being generated. Electrical consumption is low, making such units suitable for use in extramural settings outside regular fixed dental clinics, such as in mobile facilities.

For the sterilisation of wrapped items by HPGPS, pouches are made with high-density spun-bound polyethylene fibers (DuPont Tyvek) on one side and a polyethylene/polyester lamination on the other side. A low-temperature heat sealer is used to seal the pouches. Tyvek pouches cannot be used with steam sterilisation because of their low melting point.

HPGPS sterilisation is not suitable for any items that can absorb liquid hydrogen peroxide, including cellulose (paper) and cotton (eg, cotton rolls). It is necessary to use chemical indicators and biological indicators (spore tests) specifically designed for HPGPS, and these are readily available.^47^, ^48^, ^49^ As with steam sterilisation, it is necessary to record key data for each cycle and for staff to check the data for each cycle at the completion of each sterilisation cycle, in line with local policies and regulations.

A key gap in knowledge regarding HPGPS is its overall environmental impact when compared to steam sterilisation. The considerations include the costs to manufacture the 2 different types of sterilisers, as well as their running costs (electricity, hydrogen peroxide or deionised water, servicing costs, useable life), the differential costs of packaging in Tyvek pouches vs other materials, and the waste outcomes for the packaging materials. A life-cycle assessment should be undertaken to properly inform end users of the costs and benefits of these 2 competing approaches.

In addition, a body of work is needed to verify the optimal protocols for using HPGPS sterilisation for sterilising heat-sensitive plastic items designed for sterilisation using steam. In addition, the potential for the same approach to be used with other plastic items must be explored because this will be required to inform manufacturers and alter clinical guidelines on reprocessing once the necessary regulatory approvals have been granted. The overall goal is to reduce the volume of items that are currently single-use disposable plastic items, without compromising on the requirements for effective reprocessing.

### Disposable instruments

In some parts of the world, disposable dental instruments are readily available, ranging from examination kits (with a dental mirror and probes) to sterile extraction forceps. Some dental services use disposable sterile instruments to avoid risks associated with errors in sterilisation or to lower operational costs and complexity by avoiding the need for “back-to-base” instrument reprocessing altogether.

Provided that reprocessing of reusable instruments includes careful checking for corrosion and wear, as well as sharpness and functionality, the in-use or operational performance of reusable surgical instruments should be no less than that of disposable singe-use surgical instruments. On the other hand, disposing of single-use surgical instruments into waste streams that go to landfills or to incineration generates other issues related to the environment.

Life-cycle analyses have revealed that disposable instruments generate greatly increased greenhouse gas equivalents when compared with reusable alternatives, owing to higher manufacturing and transport costs.^50^ Regular reusable surgical instruments, on the other hand, incur one-off manufacturing costs, along with recurring costs for reprocessing over the life of the instrument. A regular surgical instrument of good quality, if properly cared for in clinical use and during reprocessing, would be expected to last 10 years.^51^ This provides a useful benchmark when making decisions related to disposable instruments.

## Conclusions

The platform concepts behind decontamination of environmental surfaces and the reprocessing of dental instruments have remained unchanged for many years, and key principles such as cleaning before disinfection or sterilisation remain true to the present day. What has changed in recent years are the range of options available, most notably for no-touch decontamination as an adjunct to regular cleaning methods, and alternatives in the realm of sterilisation, including solar autoclaves and HPGPS. As an overlay across all such developments, there is now greater attention to safety considerations for staff and a stronger emphasis on environmental impacts. Since dental clinics have many configurations and sizes, each clinic must undertake local facility risk assessments to inform their decisions regarding decontamination methods.

## Author contributions

Laurence Walsh assessed the literature and wrote and revised the manuscript.

## Funding

This work did not receive any specific grant from funding agencies in the public, commercial, or not-for-profit sectors.

## Conflict of interest

None disclosed.
